# Efficacy of propofol-supplemented cardioplegia on biomarkers of organ injury in patients having cardiac surgery using cardiopulmonary bypass: a statistical analysis plan for the ProMPT-2 randomised controlled trial

**DOI:** 10.1186/s13063-024-08016-w

**Published:** 2024-02-29

**Authors:** Helena Smartt, Gianni D. Angelini, Ben Gibbison, Chris A. Rogers

**Affiliations:** 1 Bristol Trials Centre, Bristol Medical School, University of Bristol, Bristol, UK; 2 National Institute for Health Research Bristol Biomedical Research Centre, University Hospitals Bristol and Weston NHS Foundation Trust and University of Bristol, Bristol, UK; 3 Bristol Heart Institute, University of Bristol, Bristol, UK; 4 Department of Anaesthesia, University Hospitals Bristol and Weston NHS Foundation Trust, Bristol, UK

**Keywords:** Cardiac surgery, Cardiopulmonary bypass, Cardioplegia, Ischaemia, Reperfusion, Propofol, Randomised controlled trial, Statistical analysis plan

## Abstract

**Background:**

The ProMPT-2 trial (*Pro*pofol for *M*yocardial *P*rotection *T*rial #2) aims to compare the safety and efficacy of low- and high-dose propofol supplementation of the cardioplegia solution during adult cardiac surgery versus sham supplementation. This update presents the statistical analysis plan, detailing how the trial data will be analysed and presented. Outlined analyses are in line with the Consolidated Standards of Reporting Trials and the statistical analysis plan has been written prior to database lock and the final analysis of trial data to avoid reporting bias (following recommendations from the International Conference on Harmonisation of Good Clinical Practice).

**Methods/design:**

ProMPT-2 is a multi-centre, blinded, parallel three-group randomised controlled trial aiming to recruit 240 participants from UK cardiac surgery centres to either sham cardioplegia supplementation, low dose (6 µg/ml) or high dose (12 µg/ml) propofol cardioplegia supplementation. The primary outcome is cardiac-specific troponin T levels (a biomarker of cardiac injury) measured during the first 48 h following surgery.

The statistical analysis plan describes the planned analyses of the trial primary and secondary outcomes in detail, including approaches to deal with missing data, multiple testing, violation of model assumptions, withdrawals from the trial, non-adherence with the treatment and other protocol deviations. It also outlines the planned sensitivity analyses and exploratory analyses to be performed.

**Discussion:**

This manuscript prospectively describes, prior to the completion of data collection and database lock, the analyses to be undertaken for the ProMPT-2 trial to reduce risk of reporting and data-driven analyses.

**Trial registration:**

ISRCTN ISRCTN15255199. Registered on 26 March 2019.

## Introduction

Cardiac and circulatory diseases cause around a quarter of all deaths in the United Kingdom (UK) and coronary heart disease (the most common form of heart disease) was the most common cause of death worldwide in 2019 [1]. Close to 25,000 cardiac surgical operations were performed in 2021/2022 in the UK (not including Scotland) [2]. During cardiopulmonary bypass (CPB) the heart is stopped, using a solution called cardioplegia, given directly into the coronary arteries. However, the cardiac muscle can become deficient in oxygen during this time (ischaemia) and injury can occur on the return of blood flow once the heart is restarted (reperfusion injury). In the PROMPT trial, we previously reported evidence that adding a low dose of the general anaesthetic agent propofol (6 µg/ml) to the cardioplegia solution can protect the heart against this ischaemia-reperfusion injury in coronary artery bypass grafting (CABG) or aortic valve replacement surgery [3]. The ProMPT-2 trial aims to investigate the safety and efficacy of supplementing the cardioplegia with a higher concentration of propofol (12 µg/ml), in comparison with the concentrations used in the PROMPT study (6 µg/ml) and sham.

The ProMPT-2 trial is a multi-centre, parallel three-group randomised controlled trial. Participants are randomised in a 1:1:1 ratio to either (i) sham supplementation, (ii) low dose propofol (6 µg/ml) or (iii) high dose propofol (12 µg/ml). The study population is adults undergoing first time, urgent or elective CABG surgery at a specialist UK cardiac surgery centre. Full details of the study background and design have been reported elsewhere [4]. This analysis plan was written and finalised during the 12-month follow-up period for the trial and in advance of any analysis of trial outcomes to minimise reporting bias.

### Study objectives

The objectives of this trial are to estimate (a) the difference between the three groups in the cardiac-specific troponin T (cTnT) response after surgery (as a biomarker of cardiac injury) and whether high-dose propofol reduces cTnT more than low-dose propofol; (b) the difference between groups with respect to a range of other biomarkers (e.g. of renal injury, inflammation, oxidative stress and metabolic stress); (c) the frequencies of serious adverse events, and serious adverse reactions in the three groups and (d) the difference between groups in Quality of Life (QoL) at 3 and 12 months following surgery. The study obtained research ethics approval from South Central – Berkshire B Research Ethics Committee and Medicines and Healthcare products Regulatory Agency (IRAS ref: 234266) and is registered with *ClinicalTrials.gov* (ISRCTN15255199).

### Outcomes

The primary outcome is myocardial injury, assessed through serial measurement of cardiac-specific Troponin T (cTnT) in serum from blood samples collected pre-operatively and during the first 48 hours after chest closure.

Secondary outcomes include:

1. Systemic metabolic stress, as measured by blood lactate (mmol/L)
2. Renal function, as measured by serum creatinine (µmol/L)
3. Markers of inflammation and oxidative stress as measured by interleukin (IL)-6, IL-8, IL-10, tumour necrosis factor (TNF)-α (in pg/mL) and myeloperoxidase (MPO, in ng/mL) in serum (measured in one trial site only)
4. Blood pH
5. Length of intensive care unit (ICU) stay (from end of surgery to ICU discharge, days)
6. Length of postoperative hospital stay (from end of surgery to hospital discharge, days)
7. Clinical outcomes and serious adverse events, i.e. serious post-operative complications (e.g. myocardial infarction, permanent stroke, acute kidney injury) and death from any cause
8. QoL measured with the Coronary Revascularisation Outcome Questionnaire (CROQ) and the EQ-5D-5L questionnaire (CROQ core total, EQ-5D utility score)

The primary outcome and secondary outcomes 1 to 4 above are measured pre-operatively and at 1, 6, 12, 24 and 48 h post-operatively (from knife down time). An additional measurement is made for secondary outcome 1 (blood lactate) at 10 min after cross-clamp release. Clinical outcome data will be collected during the post-operative hospital stay: only deaths will be collected during follow-up after hospital discharge. Quality of Life data are collected pre-operatively and at 3 months and 12 months post-operatively.

### Sample size

Based on previous data [3] we assumed correlations between the pre- and post-operative cTnT measurements of 0.2 and between successive post-operative measures of 0.7. With 5 repeated post-operative measurements and these correlations, a sample size of 240 participants (80 per group) will provide 90% power at a 5% significance level (2-sided) to detect a difference in cTnT of 0.25 standard deviations (SD) between adjacent groups (i.e. a 0.25 SD difference between placebo and low dose propofol and between low and high dose propofol respectively), when all groups are considered together in one overall analysis. This allows for a 7.5% dropout rate. This sample size also gives 90% power to detect a moderate difference of 0.5 SD (the target difference in the PROMPT study, [3]), between any two groups at a 1.67% significance level.

### Flow of participants

**Fig. 1:**
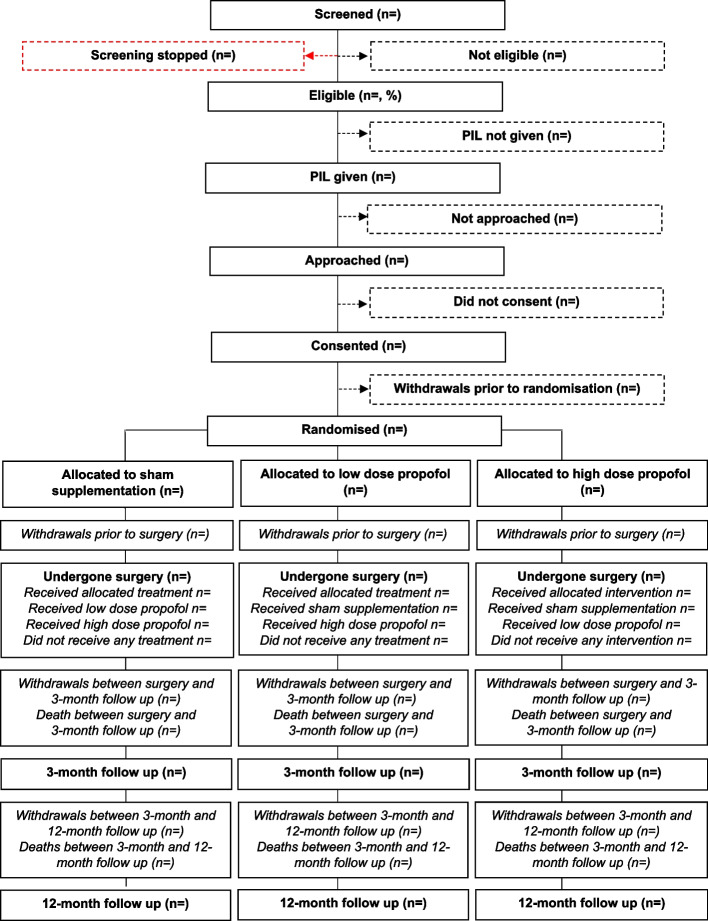
Participant flowchart

Participant flow through the study will be described using a flowchart as recommended by the Consolidated Standards of Reporting Trials (CONSORT) [5] (Fig. 1). Screened patients fulfilling all eligibility criteria and giving written informed consent are randomised as close to the start of surgery as possible. Participants are allocated in a 1:1:1 ratio to (i) high-dose propofol, (ii) low-dose propofol or (iii) sham supplementation using computer-generated allocation sequences stratified by site with random block sizes.

### Withdrawals/changes in participation status

Participants may withdraw their consent to participate in the trial at any time, requesting the cessation of routine data collection while in hospital and/or participation in follow-up. A clinician may withdraw a participant from receiving the trial treatment at any time if judged to be in the participant’s best interest. Where possible, data collection will continue after a change in trial participation status, regardless of whether the participant received the allocated trial treatment. The timing, reasons and details surrounding the change in participant status will be described by treatment allocation.

### Adherence with study protocol

Non-adherence with the trial protocol (including manual randomisations, randomisation of ineligible participants, failure to receive the allocated treatment, departures from the anaesthesia and myocardial protection procedures given in the protocol and any notable breaches of good clinical practice) will be reported. The frequency of each type of protocol non-adherence will be described by treatment allocation.

### Analysis population

All statistical analyses will be conducted on a modified intention to treat (mITT) population unless otherwise specified. The mITT population will consist of all randomised participants, excluding participants withdrawn who were unwilling for data collected to be used or participants with no post-operative outcome data, e.g. due to withdrawal. Participants will be grouped according to their allocated treatment (except for reporting of harms: see ‘Safety data’ section), regardless of whether they are ineligible, prematurely discontinue treatment or are otherwise protocol deviators.

## Statistical analysis principles/general statistical considerations

### General considerations

Analysis principles and data presentation will follow the guidance issued in the CONSORT statement [5] and will be carried out in Stata version 17.0. Likelihood ratio tests will be used in hypothesis testing in preference to Wald tests. All statistical analysis of study outcome data will be performed after completion of follow-up and database lock.

### Modelling considerations including multiplicity

Where treatments are being compared using statistical modelling, the sham supplementation group will act as the reference category and all applicable statistical tests will be 2-sided superiority comparisons. For each outcome to be compared statistically, a model with indicators for the two propofol groups (i.e. assuming no ordering to the groups) will be compared to a model which assumes an ordinal linear dose response relationship with increasing dose of propofol supplementation (i.e. as hypothesised, with the difference between the sham supplementation and low dose being the same as the difference between the low and high doses). If there is not a statistically significant difference in model fit between the two models at the 5% level, the ‘dose response’ model will be chosen (and we will not investigate the ‘unordered group’ model further). Conversely, if there is a statistically significant improvement in fit using the ‘unordered group’ model then this will be selected. An overall assessment of the effect of treatment across the three groups will be quantified, and differences between pairs of treatments (e.g. sham vs low dose supplementation, low vs high dose supplementation and sham vs high dose supplementation) will be reported, using 98% CIs and a 1.67% significance level to take into account multiplicity associated with three outcome comparisons. Otherwise, comparisons will be performed using a 5% significance level, apart from tests for interactions which will be performed using a 10% significance level (see the ‘Outcome data analyses’ section), and confidence intervals will be 95%. There will be no further adjustment for multiple testing, though consideration will be taken in interpretation of results of the number of statistical tests performed and the direction, magnitude, and consistency of treatment estimates for different outcomes.

### Descriptive analyses

Pre-randomisation characteristics (e.g. participant demography and baseline data), intra-operative details and relevant post-operative information will be described by treatment allocation for the analysis population. Participants who withdrew from the trial but allowed their data to be used will be included. Categorical data will be summarised as a number and percentage and continuous data will be summarised using the mean and SD (or median and inter-quartile range (IQR) if the distribution is skewed). Any apparent imbalances in participant characteristics will be described but statistical tests for imbalance will not be carried out, in line with recommendations [5].

### Safety data

Safety data will be reported under the secondary outcome of clinical outcomes (adverse events (AEs)) and serious adverse events (SAEs) i.e. serious post-operative complications (e.g. myocardial infarction, permanent stroke, acute kidney injury) and death from any cause. SAEs are defined as AEs that lead to or prolong an existing hospitalisation, that are life-threatening, that result in persistent or significant disability or cause death. Complications occurring post-operatively (and prior to hospital discharge) will be described by treatment received but not compared statistically due to lack of power for analysis of binary outcomes. Deaths will be collected and reported for the whole 12-month follow-up period. Detail concerning events occurring in participants that did not receive their allocated treatment will be indicated in footnotes. Medical Dictionary for Regulatory Activities (MedDRA) System Organ Class and Preferred Terms will be derived for all events using the most recent version of MedDRA at the time of analysis.

### Outcome data analyses

With the exception of clinical outcomes and serious adverse event data (see previous section), all trial outcomes described in the protocol will be compared statistically through analysis either as a time-to-event outcome (length of time in ICU and in hospital) or a continuous longitudinal outcome (cTnT, blood lactate, serum creatinine, inflammatory and oxidative stress markers, blood pH and QoL) as described below.

### Time-to-event outcomes

#### Modelling and reporting considerations

Time-to-event outcomes will be summarised using the median and IQR in each treatment group and compared using Cox’s proportional hazards (PH) models, unless the PH assumption is not met: in which case parametric models (e.g. an accelerated failure time model) may be more suitable. Treatment comparisons will be presented as hazard ratios and associated CIs if a proportional hazards model is used or e.g. time ratios and associated CIs if an accelerated failure time model is used.

#### Data handling considerations

Participants without a hospital discharge date (e.g. due to transfer to another hospital) will be censored at the time of this transfer. Participants that die before ICU or hospital discharge will be censored at the longest recorded ICU or hospital stay, respectively, as this is computationally equivalent to competing risks methodology in this setting [6].

### Continuous longitudinal outcomes

#### Reporting considerations

Continuous longitudinal outcomes will be summarised at each timepoint using the mean and SD or the median and IQR (as described in the ‘Descriptive analyses’ section) or, if a logarithmic transformation provides an approximately normal distribution, using geometric means and 95% CIs. Treatment comparisons will be presented as adjusted differences in means with associated CIs (or as geometric mean ratios and associated CIs if data are skewed).

#### Data handling considerations

Bounded continuous outcome data may be partially- or fully categorised if inflation at either of the data bounds is observed. Participants who died during the course of the trial will be assigned an EQ-5D utility score of zero for all timepoints following death [7].

#### Modelling considerations

Outcomes will be compared using linear mixed effects methodology or, if model fit is inadequate for bounded data, one- or two-part logistic modelling (of fully or partially categorised data) may be considered. Treatment group, site (where applicable) and baseline measurements will be fitted as fixed effects, and participant terms fitted as random effects. To determine whether the effect of treatment changes with time, a model including a time-by-treatment interaction term will be compared to a model without this interaction term. If time-by-treatment interaction is statistically significant at the 10% level the changes in treatment effect with time will be described; if the interaction term is not significant at the 10% level an overall treatment effect will be reported. Different variance-covariance structures will be explored (compound symmetry, first-order autoregressive, Toeplitz and unstructured) and the structure which provides the best fit (in terms of likelihood ratio tests or Akaike information criterion if non-nested models are compared) will be used.

## Adjustment in models

As randomisation was stratified by site, the intention is to adjust all models for site (except when modelling inflammatory and oxidative stress data, which was only collected for one of the trial sites) through fitting site as a fixed effect or, for survival models, through stratifying by site. Pre-randomisation measures of continuous longitudinal outcomes will also be included as covariates in the analysis models.

## Model assumptions

For all methods described above, the underlying assumptions will be checked using standard methods e.g. residual plots, tests for proportional hazards etc. If assumptions are not valid then alternative analysis methods will be sought (e.g. by applying a logarithmic transformation to positively skewed continuous data). If outlying observations are identified which mean that models do not fit the data adequately, sensitivity analyses omitting such observations may be considered.

## Missing data

Missing data will be minimised by performing a thorough data cleaning process until data are either received, confirmed as unavailable or the trial has reached the analysis stage. Missing data will be indicated in all tables using observation counts for categorical variables and indicated by footnotes for continuous variables. If the amount of missing data differs substantially between treatment groups, then potential reasons will be explored (reasons for missing outcome data will be included in outcome data tables where known).

In terms of predictors, there will be no missing data for any of the randomisation factors (i.e. site) by design. All remaining potential predictors are pre-randomisation measurements of continuous longitudinal outcomes. If missing for less than 5% of participants, these missing values will be imputed using mean or median values (depending on the distribution of non-missing data) for the whole cohort. If missing for more than 5% of participants, then multiple imputation will be considered.

Because longitudinal models use all available data across all time points, there is little benefit in imputing missing postoperative outcome data if the missing data can be assumed to be missing at random (MAR; [8]). The assumption that data are MAR will be assessed by comparing the variances of continuous outcomes across the groups and the dropouts/withdrawals in each group: if these are similar then the data can be assumed to be MAR [9]. It is expected that outcomes are more likely to be missing if the participant has a ‘poor’ outcome, but it is not anticipated that missingness will be related to the treatment group. If the MAR assumption does not hold and missingness/dropout is not balanced across groups, imputation methods which do not assume MAR will be considered [10].

It is anticipated that some data for cTnT and markers of inflammation and oxidative stress will be outside the assay detection range and imputation of alternative values for these data will be considered. For example, where the lower detection limit is 5 ng/mL, using the midpoint between 0 and 5 if the number of values below 5 is few (< 5%) or using multiple imputation between the values of 0 and 5 if more than 5% are below 5. For baseline (pre-operative) values below the detection limit a categorisation of the values (e.g. < 5, 5–10 etc.) may also be considered if a large proportion (> 20%) of the data are below the lower detection limit, with cut points chosen to give approximately equal observations in each category above the detection limit. Similarly, if post-operative values above the upper limit of assay detection are observed, these will be imputed. For example, the upper detection limit of the cTnT assay is 10,000 ng/L: if < 5% of observations are > 10,000 then a value of 10,000 will be used and if > 5% are > 10,000 then multiple imputation of values > 10,000 will be considered.

For multiple imputation, a general imputation model using an iterative procedure to generate imputed values will be used to generate multiple complete data sets (e.g. using Stata’s mi impute). The model of interest would then be fitted to each complete dataset and effect estimates combined using Rubin’s rules. If appropriate, methods such as predictive mean matching will be used to ensure that imputed values lie within specific ranges.

## Sensitivity analyses

A number of sensitivity analyses are proposed to investigate the robustness of the main trial analyses. Two sensitivity analyses will be performed for the primary outcome to address the impact of:-

1. Major deviations from the trial protocol, by including only eligible participants who received one form of trial treatment. The analysis will be performed according to the treatment received.
2. Excluding measurements that were ‘out of window’ (e.g. a 12-h measurement taken at 19 h, so closer to the 24-h time point that the intended 12 h or taken at 8 h, so closer to the 6-h time point that the intended 12 h)

Assays to assess levels of inflammatory and oxidative stress are performed in duplicate (as there is insufficient funding to allow all assays to be performed in triplicate). In the primary analysis of these outcomes, the two replicates will be included in the model. A third repeat assay is carried out if the two measurements are not in agreement (i.e. the values differ by more than a pre-defined threshold, guided by previous experience and reported assay coefficient of variation). These analyses will include the results of the third assay and a variable indicating whether data were from the original run of the samples or from a re-run.

Finally, sensitivity analyses omitting outlying observations may be considered (see 'Model assumptions’ section).

## Pre-specified ancillary analyses

Several ancillary analyses are proposed to investigate the impact of the COVID-19 pandemic, which commenced during trial recruitment, on the trial population/demographics, selected outcomes, and follow-up rates. Demographic data and follow-up rates will be tabulated separately for (a) participants recruited before or during and (b) after the COVID-19 pandemic, using 19/7/2021 (lifting of the final stage of COVID-19 restrictions in England) as the pandemic end date. Participants recruited before or during the pandemic will be grouped together because of relatively low numbers recruited prior to the start of the pandemic and because very few of these pre-pandemic recruits completed their 12-month follow-up prior to the start of the pandemic. If these descriptive analyses suggest that the COVID-19-related cohorts differ, then the primary outcome analysis will be repeated using an interaction between the trial treatment and the COVID-19 cohort to determine whether the effect of the trial treatment differed between the COVID-19 cohorts. Overall QoL scores will also be summarised by the COVID-19 cohort and an exploratory analysis will be carried out adjusting each QoL score regression model for the COVID-19 cohort.

## Changes to the original analysis plan

A basic analysis plan was written as part of the trial protocol, version 9 of which has been followed when writing the current detailed statistical analysis plan, with some additions made. These include details of:

- Considerations of multiplicity/multiple testing,
- Complete list of variables to adjust for in analysis models,
- Further details of analysis modelling approach, including censoring variables for time-to-event analyses, and alternative approaches if fit of planned models is inadequate or model assumptions are violated,
- Strategy for dealing with missing data and data beyond assay limits,
- Pre-specified sensitivity and ancillary analyses, and
- Planned content of trial output including CONSORT flow diagram.

Two secondary outcomes were removed from version 9 of the trial protocol due to becoming logistically and financially unfeasible during the trial. These were mechanistic microRNA sub-studies investigating the association between cTnT and circulating levels of cardiac-released microRNA-1 and exosomal microRNA-1 content, and whether this differed between trial treatment groups. As a result, these outcomes are not covered by this Statistical Analysis Plan.

## Discussion and trial status

Following on from the publication of the trial protocol [4], here we prospectively detail the statistical analysis plan to evaluate the ProMPT-2 trial, in advance of any outcome data analysis. This will help to reduce the risk of reporting bias and data-driven analyses.

This article was compiled with reference to “Guidelines for the Content of Statistical Analysis Plans in Clinical trials” [11].

240 participants from three sites have been recruited, meeting the target sample size. The Statistical Analysis Plan (version 1) was signed off in October 2023, prior to database lock and any statistical analysis of study outcome data. At the time of writing, the trial is in the follow-up phase, with trial follow-up due to finish in November 2023.

## Data Availability

Not applicable.

## Abbreviations

AE: Adverse event
CABG: Coronary artery bypass graft
CI: Confidence interval
CONSORT: Consolidated Standards of Reporting Trials
CPB: Cardiopulmonary bypass
CROQ: Coronary Revascularisation Outcome Questionnaire
cTnT: Cardiac-specific troponin T
CTU: Clinical trials unit
COVID-19: Coronavirus disease 2019
EUDRACT: European Union Drug Regulating Authorities Clinical Trials
EME: Efficacy and Mechanism Evaluation
EQ-5D: EuroQol 5-Dimension
ICU: Intensive care unit
IL: Interleukin
IRAS: Integrated Research Application System
ISRCTN: International Standard Randomised Clinical Trial Number
MAR: Missing at Random
MedDRA: Medical Dictionary for Regulatory Activities
mITT: Modified intention to treat
MPO: Myeloperoxidase
MRC: Medical Research Council
NIHR: National Institute for Health and Care Research
PH: Proportional hazards
RNA: Ribonucleic acid
QoL: Quality of life
REC: Research Ethics Committee
SAE: Serious adverse event
SD: Standard deviation
TNF-α: Tumour necrosis factor alpha
UK: United Kingdom
